# Chitosan‐Based Hydrogels for Periodontal Therapy: From Drug‐Polymer Chemistry to Smart Multifunctional and Regenerative Systems

**DOI:** 10.1002/cbdv.71891

**Published:** 2026-10-31

**Authors:** Vikesh Kumar, B. T. Nandish, Sowmya Rao, Manojna K. M.

**Affiliations:** ^1^ Department of Dental Materials Yenepoya Dental College, Yenepoya (Deemed to Be University) Mangaluru Karnataka India

## Abstract

Periodontitis is a chronic, multifactorial inflammatory disease driven by microbial dysbiosis, oxidative stress, and dysregulated host immune responses, resulting in progressive destruction of periodontal tissues and, ultimately, tooth loss. Traditional treatments, including scaling and root planing and systemic antibiotics, have limitations due to incomplete bacterial elimination, poor site specificity, short drug retention, systemic side effects, and limited regenerative support. Recently, chitosan (CS)‐based hydrogels have emerged as promising biomaterials for localized periodontal treatment owing to their biocompatibility, biodegradability, mucoadhesive nature, and ease of chemical modification. This review critically discusses recent developments in CS‐based hydrogel systems functionalized with antibiotics, phytochemicals, anti‐inflammatory agents, nanoparticles, regenerative molecules, and stimuli‐responsive materials for the management of periodontitis. It primarily focuses on periodontal pathophysiology, therapeutic strategies, and drug‐polymer interactions that affect its performance. Furthermore, it highlights emerging smart hydrogel systems that respond to pH, reactive oxygen species, temperature, and enzymatic activity within the periodontal microenvironment for controlled, targeted drug delivery. Future perspectives involve the development of multi‐stimuli‐responsive, microbiome‐preserving, self‐healing, and personalized hydrogel platforms capable of sequential drug delivery and periodontal regeneration. On the whole, this review bridges the gap between materials chemistry and clinical applicability, providing insights into design of next‐generation CS‐based hydrogels for targeted periodontal therapy.

## Graphical abstract

Chitosan‐based hydrogels are evolving from simple local drug carriers into multifunctional periodontal platforms that integrate antimicrobial, anti‐inflammatory, antioxidant, and regenerative functions. Chemical crosslinking, nanocomposite design, and stimulus‐responsive architectures enable adaptive therapeutic delivery. However, standardized evaluation, long‐term safety, manufacturability, and clinical benchmarking remain essential for advancing these systems toward clinical application.

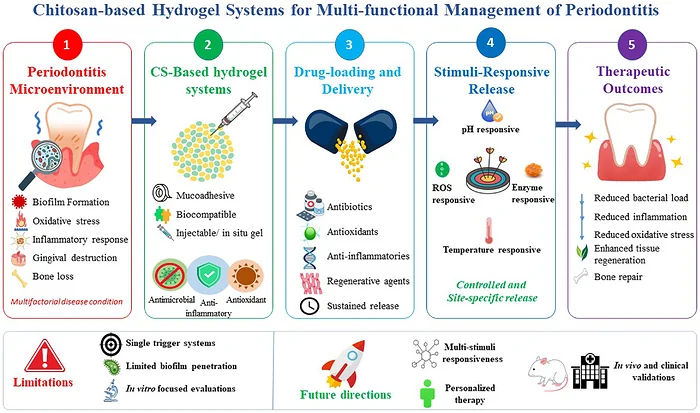

## 1 Introduction

Periodontitis is a chronic inflammatory disease characterized by progressive destruction of the periodontal ligament, cementum, and alveolar bone, ultimately leading to tooth loss if left untreated [1]. The disease is initiated by dysbiotic polymicrobial biofilms harboring pathogenic species such as *Porphyromonas gingivalis*, *Treponema denticola*, and *Tannerella forsythia*, which trigger persistent inflammatory and oxidative stress responses in periodontal tissues [2, 3]. In response to these increasing levels of reactive oxygen species (ROS), inflammatory cytokines, and proteolytic enzymes contribute to collagen degradation, alveolar bone resorption, and periodontal tissue destruction [1, 4].

Although conventional approaches such as scaling and root planing, systemic antibiotics, surgical procedures, and photodynamic or laser treatments remain widely used, their long‐term effectiveness are limited. Challenges such as incomplete biofilm removal, inadequate drug retention within periodontal pockets, systemic side effects, and the complex nature of the disease can reduce treatment outcomes. These limitations have encouraged the development of advanced drug delivery systems capable of providing sustained drug release while responding to changes in the periodontal microenvironment [5, 6].

Among the various biomaterials investigated, hydrogels are emerging as promising candidates for periodontal drug delivery due to their exceptional biocompatibility, high water‐retention capacity, injectability, and biomimetic properties [7, 8]. In particular, chitosan (CS)‐based hydrogels are gaining attention owing to their biodegradability, mucoadhesiveness, antimicrobial activity, and ease of chemical functionalization. The presence of amino (─NH_2_) and hydroxyl (─OH) groups enables functional group modification, dynamic crosslinking, and the incorporation of multiple therapeutic agents for controlled, stimuli‐responsive drug delivery. Recent developments are increasingly focusing on multifunctional, smart CS‐based hydrogels that can respond to pathological stimuli in periodontal pockets, such as low pH, high ROS levels, enzymatic activity, and inflammatory conditions [9, 10, 11]. These systems combine host‐modulatory and regenerative effects into a single therapy, moving from passive drug delivery to precise periodontal treatment.

This review critically discusses recent developments in CS‐based hydrogels for managing periodontitis, focusing particularly on drug‐polymer chemistry, multifunctional therapeutic strategies, regenerative systems, and stimuli‐responsive smart hydrogels. Furthermore, the review evaluates the ideal physicochemical and biological characteristics required for effective intra‐pocket delivery, highlights current translational limitations, and outlines the future perspectives for the development of clinically relevant adaptive periodontal biomaterials.

## 2 Literature Search Strategy

Literature included in this review was accessed from PubMed, Scopus, Web of Science, and Google Scholar databases using the keywords such as “chitosan,” “periodontitis,” “stimuli‐responsive hydrogel,” “nanoparticles,” “periodontal regeneration,” and “injectable hydrogels.” Studies published between January 2020 and 7 September 2026 were considered, including both preclinical and clinical studies investigating antimicrobial, anti‐inflammatory, regenerative, and stimuli‐responsive CS‐based systems for periodontal applications. The final literature search was conducted on 7 September 2026. Original studies focusing on CS‐based hydrogels were included as the primary evidence. At the same time, relevant non‐hydrogel CS formulations (e.g., nanoparticles, membranes, films, chips, and scaffolds) were retained only for contextual comparison. Conference abstracts, book chapters, non‐English articles, and studies lacking direct relevance to periodontal medicine were excluded. Duplicates were identified and removed using the Zotero reference‐management system. Records were screened by the first author and independently reviewed by the second author, with discrepancies resolved through consensus; the remaining authors contributed to critical appraisal and synthesis of the literature.

## 3 Periodontal Microenvironment and Disease Pathophysiology

Periodontitis is a multifactorial inflammatory disease in which microbial dysbiosis, an imbalanced host immune response, and oxidative stress contribute to the progressive destruction of periodontal tissues. The periodontal pocket represents a dynamic and challenging microenvironment, where local pH can become acidic depending on disease severity, the composition of the subgingival microbiota, and gingival crevicular fluid turnover. Increased ROS, along with inflammatory cytokines and enzymes released in response to microbial activity, thereby creating a hypoxic condition [3, 4].

This shift from healthy gums to periodontal disease is associated with the transition from symbiotic microbial communities towards the pathogenic anaerobic species, particularly the red complex bacteria, including *Porphyromonas gingivalis*, *Treponema denticola*, and *Tannerella forsythia* [2]. These organisms produce virulence factors, such as lipopolysaccharides, proteases, and metabolic by‐products, that stimulate host immune responses, promote inflammatory tissue destruction, and promote disease progression.

One of the major issues in periodontitis is excessive ROS generation. Although ROS initially act as defensive molecules against bacterial colonization, continued oxidative stress leads to collagen degradation, lipid peroxidation, osteoclast activation, and alveolar bone resorption [4]. Simultaneously, increased levels of inflammatory substances such as TNF‐α, IL‐1β, IL‐6, and matrix metalloproteinases will further worsen the periodontal tissue breakdown and hinder regenerative healing.

The periodontal microenvironment poses several challenges for effective drug delivery. Continuous salivary flow, gingival crevicular fluid turnover, masticatory forces such as chewing and swallowing, tongue movements, and other mechanical stresses can reduce drug retention within the periodontal pocket and consequently limit therapeutic efficacy. In addition, the acidic and enzyme‐rich conditions may affect polymer stability, potentially causing premature drug degradation and making it difficult to maintain effective drug concentrations at the site of infection [9, 10].

Hence, effective periodontal materials should not only provide sustained antimicrobial activity but also respond intelligently to pathological conditions such as acidic pH, oxidative stress, enzymatic activity, and inflammatory signaling. This has led to growing interest in multifunctional CS‐based hydrogel systems with combined bioactive functions [11]. The progression of periodontitis, associated pathological microenvironment, and the major therapeutic strategies employed in CS‐based hydrogel systems are illustrated in Figure 1.

**FIGURE 1:**
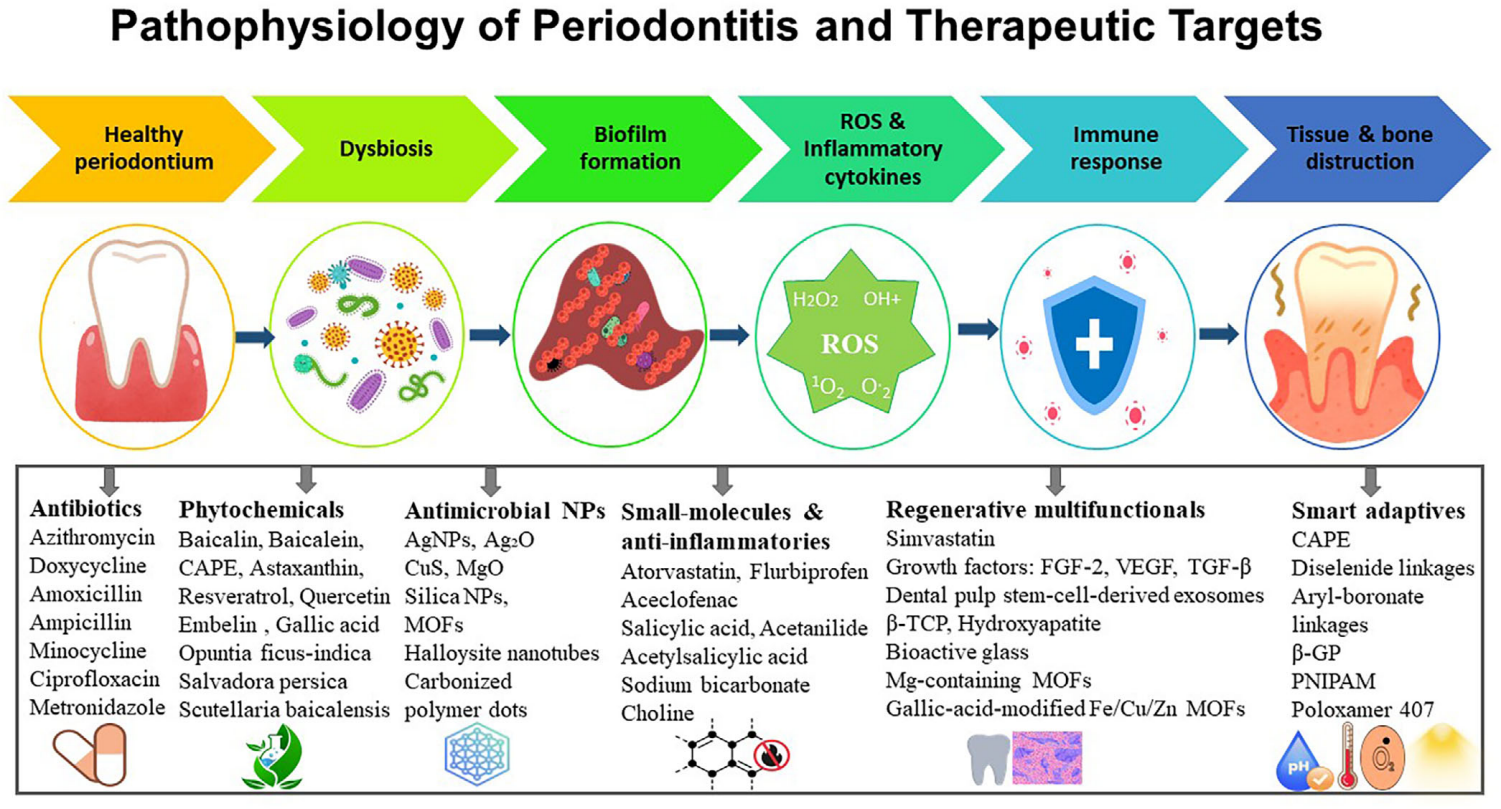
Schematic representation of the pathophysiology of periodontitis and therapeutic targets of chitosan‐based hydrogel systems. Abbreviations: AgNPs, silver nanoparticles; Ag2O, silver oxide; CAPE, caffeic acid phenethyl ester; CuS, copper sulfide; FGF‐2, fibroblast growth factor‐2; MgO, magnesium oxide; MOFs, metal–organic frameworks; PNIPAM, poly(N‐isopropylacrylamide); ROS, reactive oxygen species; TGF‐β, transforming growth factor‐beta; VEGF, vascular endothelial growth factor; β‐GP, β‐glycerophosphate; β‐TCP, beta‐tricalcium phosphate.

## 4 Chitosan Chemistry and Hydrogel Engineering

CS is a naturally derived cationic polysaccharide produced by the partial deacetylation of chitin. Its backbone is composed of β‐(1→4)‐linked D‐glucosamine and N‐Acetyl‐D‐glucosamine units, with reactive amino and hydroxyl groups that facilitate chemical modification and hydrogel network formation [12]. Key structural characteristics, including the degree of deacetylation, molecular weight, crystallinity, and chain architecture, play a key role in determining the physicochemical and biological properties of CS. These characteristics, in turn, influence its solubility, mucoadhesiveness, swelling behavior, enzymatic degradation, and drug‐release profile [13].

One notable aspect of CS is that it undergoes pH‐dependent protonation of its primary amine groups whenever the local environment falls below its pKa (∼6.5); this is not a fixed physiological state but varies with local pH, resulting in a positively charged polymeric backbone (─NH_2_ to ─NH_3_^+^), facilitating a strong electrostatic interaction with negatively charged biological interfaces such as mucosal tissues, bacterial membranes thereby increasing their mucoadhesiveness and antimicrobial nature. This also enables the formation of polyelectrolyte complexes for controlled and targeted drug delivery [8, 13].

CS‐based hydrogels can be developed through either physical or chemical crosslinking strategies. Physical crosslinking relies on non‐covalent interactions, including ionic interactions, hydrogen bonding, hydrophobic interactions, and thermally induced sol–gel transitions. In contrast, chemical crosslinking involves the formation of covalent bonds through mechanisms such as Schiff base reactions, genipin‐mediated crosslinking, boronate ester formation, and other covalent interactions. By selecting an appropriate crosslinking approach, the properties of the resulting hydrogel, including mechanical strength, porosity, injectability, degradation rate, and responsiveness to environmental changes, can be tailored to meet specific therapeutic requirements [12]. The structural features, functional properties, and major crosslinking mechanisms involved in CS‐based hydrogel design are summarized in Figure 2.

**FIGURE 2:**
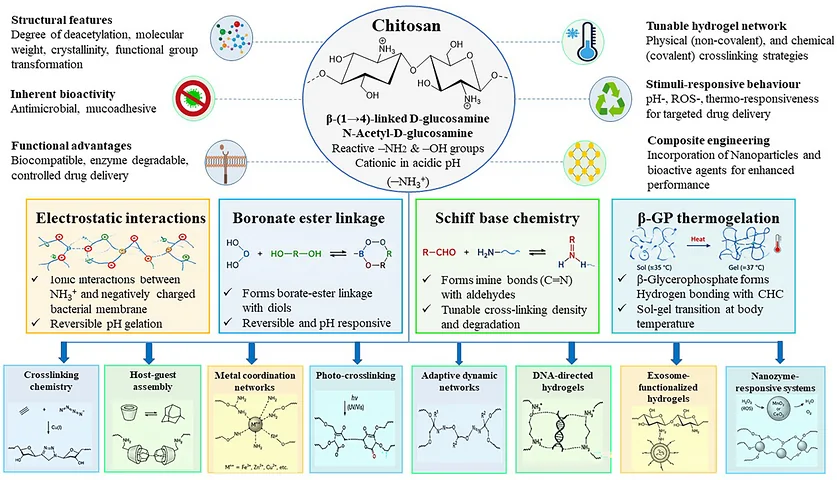
Chemical structure, functional properties, and crosslinking mechanisms of chitosan‐based hydrogels for periodontal therapy. Abbreviations: CHC, chitosan; ROS, reactive oxygen species; β‐GP, β‐glycerophosphate; NH_2_, amino group; OH, hydroxyl group; NH_3_^+^, protonated amino group.

These design strategies have also encouraged the development of stimuli‐responsive CS hydrogels that can adapt to changes in the pathological microenvironment. In periodontal applications, pH‐responsive systems take advantage of the protonation and deprotonation of CS, which can alter hydrogel swelling and promote controlled drug release in response to local pH changes. Similarly, ROS‐responsive hydrogels can incorporate oxidation‐sensitive linkages, such as boronate ester bonds, to facilitate therapeutic release under inflammatory conditions [14]. Thermoresponsive formulations, commonly incorporating β‐glycerophosphate (β‐GP), offer another approach by enabling in situ gelation through a sol–gel transition at physiological temperature [15].

The incorporation of inorganic nanomaterials and bioactive compounds has further expanded the functional capabilities of CS‐based hydrogels. Composite formulations containing copper sulfide (CuS), hydroxyapatite, bioactive glass, graphene oxide, metal oxide nanoparticles (NPs), and growth factor‐loaded carriers have shown improvements in mechanical strength, antibacterial activity, and osteoinductive properties, supporting periodontal tissue regeneration [16, 17, 18]. By combining the complementary properties of polymeric and inorganic components, these hybrid systems can provide multiple therapeutic functions that may be difficult to achieve with a single material.

Despite its versatility, CS also has some inherent limitations that need to be considered when designing hydrogel systems. Its limited solubility at physiological pH can restrict processing and in vivo applications, while variations in physicochemical properties between batches may affect reproducibility. In addition, hydrogels prepared from native CS often have relatively low mechanical strength. Therefore, CS is frequently combined with other polymers or inorganic fillers to improve structural stability and achieve the properties required for effective clinical use [11, 12, 13].

Overall, the molecular versatility of CS, together with advances in crosslinking chemistry, stimuli‐responsive design, and composite engineering, provides a strong foundation for developing multifunctional hydrogels suited to the complex periodontal environment. A better understanding of how CS chemistry, hydrogel network architecture, and biological interactions influence one another will be essential for translating these materials from experimental systems into clinically relevant periodontal therapies.

## 5 Chitosan Hydrogels as Pharmaceutical Carriers and Scaffolds

### 5.1 Mechanisms of Chitosan Hydrogel Action in Periodontal Therapy

CS hydrogels can provide several therapeutic effects in the periodontal pocket, depending on the properties of the hydrogel formulation and the agents incorporated. A single formulation often engages more than one of these mechanisms simultaneously. Antimicrobial action arises predominantly from the cationic, protonated amine groups of CS itself (see Section 4), which disrupt negatively charged bacterial membranes, together with any loaded antibiotic, antimicrobial peptide, or NPs payload that adds a second, agent‐specific mode of bacterial killing [7, 16, 17, 18, 19, 20, 21, 22, 23, 24, 25, 26, 27, 28]. Antioxidant activity is contributed either by CS itself, which has intrinsic radical‐scavenging capacity, or by incorporated phytochemicals (e.g., baicalein, astaxanthin, resveratrol, Caffeic acid phenethyl ester (CAPE)) and ROS‐responsive linkages that consume and respond to locally elevated reactive oxygen species [14, 29, 30, 31]. Anti‐inflammatory activity is achieved mainly through small molecules (e.g., flurbiprofen, aceclofenac, salicylates) and phytochemicals that suppress proinflammatory cytokine signaling, thereby reducing host‐driven tissue destruction rather than acting on the pathogen [14, 29, 30, 32, 33, 34]. Pro‐regenerative activity, distinct from the above, relies on osteogenic/angiogenic agents, bioactive inorganic components, and growth‐factor delivery to support periodontal tissue regeneration rather than simply preventing disease progression [17, 18, 27, 35, 36, 37, 38]. Several advanced systems discussed in this review (Sections 5.1.5 and 5.1.6) combine two or more therapeutic mechanisms within a single hydrogel. The following sections (Sections 5.1.1–5.1.6) are organized by therapeutic agent class to facilitate comparison of these systems and their reported biological effects.

#### 5.1.1 Antibiotics

Antibiotic‐loaded CS‐based hydrogels have been extensively investigated for localized periodontal therapy because they can improve drug retention within the periodontal pocket and provide sustained drug release. Several systems have incorporated broad‐spectrum antibiotics such as azithromycin [19], doxycycline [20], amoxicillin [22], minocycline [24], ciprofloxacin [25], and metronidazole [26] to target specific pathogenic bacteria associated with periodontitis. Recent studies primarily focus on improving drug encapsulation efficiency, mucoadhesion, biofilm penetration, and stimuli‐responsive drug release behavior.

Among these approaches, azithromycin‐loaded CS hydrogels have shown improved mucoadhesion and sustained drug release, which can enhance antimicrobial activity; however, the available evidence is still largely limited to in vitro studies, with relatively little in vivo validation [19]. Doxycycline‐loaded systems, including PLGA microspheres and CS xerogels, have also demonstrated prolonged drug release with reduced initial burst release, although their clinical potential has not yet been adequately investigated [20, 21]. Despite these promising findings, the potential development of antimicrobial resistance and questions regarding long‐term treatment sustainability remain important concerns. β‐Lactam antibiotics, particularly amoxicillin and ampicillin, have likewise been incorporated into CS‐based systems to improve localized antibacterial activity. For example, electrosprayed CS–hydroxyapatite matrices containing amoxicillin have demonstrated sustained drug release along with a reduction in the expression of inflammatory markers [22].

At the same time, ampicillin‐loaded polyelectrolyte complexes have improved control of drug diffusion and localized retention [23]. Although most studies in this category remain confined to in vitro assessments. Recent advances in nanocarrier‐assisted delivery have further expanded the functional performance of antibiotic‐loaded CS systems. Minocycline‐loaded mesoporous silica NPs coated with CS have demonstrated enhanced antibacterial efficacy, favorable biocompatibility, and regenerative potential [24]. Likewise, ciprofloxacin‐loaded nanocomposite hydrogels have demonstrated pH‐responsive release behavior and promoted osteoblast proliferation, suggesting combined antibacterial and osteogenic functionality [25]. In addition, metronidazole‐loaded thermosensitive hydrogels and collagen‐CS scaffolds have demonstrated effective activity against anaerobic periodontal pathogens, with sustained drug release [15, 26]. More recently, a dual‐drug CS hydrogel incorporating doxycycline and atorvastatin was developed. The optimized formulation provided sustained drug release for up to 72 h and demonstrated antibacterial activity against *P. gingivalis*, along with favorable cytocompatibility in vitro. The combination of an antimicrobial agent and a host‐modulatory drug illustrates the potential of dual‐drug CS‐based hydrogels to target microbial infection and periodontal inflammation simultaneously [39].

However, growing evidence indicates that indiscriminate use of broad‐spectrum antibiotics may disrupt the oral microbiome, potentially worsening dysbiosis and compromising long‐term periodontal homeostasis. Accordingly, future CS‐based systems should shift toward pathogen‐selective or microbiome‐modulating strategies, aligning with ecological approaches to periodontal therapy that emphasize host‐microbiome balance [6, 40]. In general, antibiotic‐loaded CS hydrogels primarily rely on diffusion‐controlled release, although this behavior can vary with the composition and structure of the hydrogel network [19]. From an antimicrobial stewardship perspective, localized antibiotic delivery is attractive because it allows the drug to be concentrated at the periodontal pocket while potentially reducing systemic exposure and the overall antibiotic burden. This targeted approach is one of the key reasons for the growing interest in local hydrogel‐based antibiotic delivery, beyond simply improving antimicrobial efficacy. Nevertheless, most of the antibiotic‐loaded formulations discussed above are still at the proof‐of‐concept stage, whether in vitro or in vivo. Although some systems, particularly those based on minocycline and doxycycline, have shown promising therapeutic potential, clinical evidence remains limited [20, 24]. Therefore, their translational potential remains limited, and further preclinical and clinical studies are needed to establish their safety and efficacy.

#### 5.1.2 Phytochemicals and Natural Extracts

Phytochemical‐loaded CS‐based hydrogels have gained increasing interest in periodontal therapy because of their broad range of biological activities, which extend beyond direct antimicrobial effects. Unlike conventional antibiotics that primarily target microorganisms, phytochemicals can provide multiple therapeutic effects simultaneously, making them particularly attractive for addressing the complex and multifaceted periodontal microenvironment. This type of periodontal formulations can broadly be classified into two categories: systems incorporating single, chemically defined phytochemicals and those containing complex plant extracts.

Single component system can generally be characterized, dosed, and standardized in a manner similar to conventional small‐molecule drugs. Flavonoids and other polyphenolic compounds have been extensively investigated in CS‐based delivery systems, with the aim of improving their local retention and therapeutic stability. CS systems incorporating baicalin and baicalein have shown potential to promote fibroblast migration and collagen synthesis while reducing inflammatory responses and providing sustained local drug delivery [29, 41]. Because oxidative stress plays an important role in periodontal tissue damage, ROS‐responsive phytochemical‐loaded hydrogels have emerged as a promising approach. An astaxanthin‐loaded, diselenium‐crosslinked CS hydrogel demonstrated effective ROS scavenging, in situ gelation, and improved tissue repair [30]. Similarly, CAPE‐functionalized hydrogels have been designed to respond to elevated ROS levels while reducing inflammatory signaling through modulation of the Nrf2/HO‐1 pathway [14]. More recently, resveratrol‐derived carbonized polymer dots incorporated into CS hydrogels have combined antioxidant, antibacterial, and self‐healing properties with pH‐responsive drug delivery, highlighting the potential of phytochemical‐based systems as multifunctional periodontal therapeutics [31].

Complex plant extracts contain multiple bioactive constituents, making their composition, batch‐to‐batch consistency, and regulatory standardization more challenging. *Opuntia ficus‐indica* extract‐loaded CS‐hydrogels improved antibiofilm activity, sustained drug release, and showed immune‐modulatory effect (reduced M1 and enhanced M2 polarization) [42]. At the same time, *Salvadora persica* (miswak)‐based CS periodontal chips have demonstrated clinical benefits, including reduced bleeding on probing, plaque index, and periodontal pocket depth, with improved clinical attachment levels compared with controls [43].

Despite these advantages, phytochemical‐based systems face several challenges that may hinder their clinical translation. Poor solubility, chemical instability, rapid degradation, and variability in active composition can affect their therapeutic performance. Although CS hydrogels can improve local retention and delivery, most of these formulations are still at the preclinical stage, with limited in vivo evidence and very few clinical studies to support their safety and effectiveness.

#### 5.1.3 Small‐Molecule Anti‐Inflammatory Systems

Small‐molecule anti‐inflammatory agents incorporated into CS‐based hydrogels have received increasing attention as an alternative approach to conventional antimicrobial therapy in periodontitis. These systems are designed to control excessive inflammation, oxidative stress, and extracellular matrix degradation, thereby addressing the host‐driven processes that contribute to periodontal tissue damage and disease progression.

Several CS‐based multifunctional formulations have also shown potential for reducing periodontal inflammation through their combined therapeutic effects. CuS‐mesoporous silica‐sulfated CS‐nanoclusters were reported to reduce the inflammatory response in a rat model of periodontitis, indicating their potential to support inflammation control together with periodontal tissue repair [44]. Similarly, thermoresponsive injectable CS/β‐GP/choline hydrogels containing salicylic acid derivatives reduce pro‐inflammatory cytokine expression (TNF‐α and IL‐1β) while inhibiting bacterial growth and alveolar bone resorption [32].

Injectable and thermosensitive systems can further improve drug retention at the periodontal site while supporting regenerative applications. Hydrogels incorporating sodium bicarbonate, bioactive glass, and acetanilide have shown good injectability, antibacterial activity, and apatite‐forming ability, indicating their potential for periodontal regeneration [45]. Similarly, quaternized CS films containing acetylsalicylic acid have demonstrated sustained drug release and localized therapeutic effects [33].

Recent advances have also explored nanostructure‐assisted delivery systems to further improve the performance of CS‐based hydrogels. For example, aceclofenac‐loaded halloysite nanotube (HNT)‐reinforced CS/β‐GP thermosensitive hydrogels have demonstrated sustained drug release, improved drug encapsulation, and temperature‐triggered gelation under physiological conditions [34]. The incorporation of HNTs can enhance drug‐loading capacity and extend localized anti‐inflammatory activity, highlighting the potential of nanotube‐reinforced CS systems for controlled periodontal therapy. However, further in vivo and clinical studies are needed to determine their long‐term therapeutic efficacy, biodegradation behavior, and biosafety.

Despite these promising results, most of the above current systems depend on passive drug release, and they often fail to consider the evolving nature of periodontal inflammation.

#### 5.1.4 Nanoparticles and Hybrid Systems

Antimicrobial NPs‐integrated CS‐based hydrogels have emerged as advanced multifunctional platforms for periodontal therapy, offering enhanced antibacterial efficacy, improved biofilm disruption, and additional regenerative potential. Compared with conventional antibiotic‐loaded systems, these nanocomposites provide superior local retention, sustained release, and microenvironment‐responsive therapeutic activity.

Hybrid systems incorporating antimicrobial peptides and inorganic NPs have demonstrated effective suppression of key periodontal pathogens. For instance, CS hydrogels loaded with Nal‐P‐113 peptides and polydopamine NPs exhibit combined ROS‐scavenging and antibacterial activity against *P. gingivalis* and *Fusobacterium nucleatum* with release kinetics observed up to 13 days [27].

Similarly, CuS‐mesoporous silica‐sulfated CS‐nanoclusters system increased oxidative stress in *F. nucleatum* through Cu^2^^+^‐mediated ROS signaling, thereby inhibiting bacterial growth and biofilm formation. In a rat model of periodontitis, it also reduced inflammation and promoted alveolar bone regeneration, demonstrating the potential of this CS‐functionalized nanoplatform to address both infection and periodontal tissue damage [44]. Another recent nanohydrogel strategy combined minocycline‐loaded dendritic nanogels with baicalin in a CS base. The system provided sustained minocycline release while the combined formulation exhibited antibacterial and immunomodulatory activity, including suppression of proinflammatory M1 macrophage polarization. In experimental periodontitis, they reduced alveolar bone loss and improved periodontal tissue repair, thereby demonstrating the potential of nanostructured CS hydrogels for simultaneous antimicrobial activity and modulation of the host response [28].

Metal‐based NP systems further expand the functional scope of CS hydrogels. CuS NP‐loaded injectable hydrogels generate ROS‐mediated antibacterial effects, also promoting angiogenesis and alveolar bone regeneration [16]. Although silver‐based systems exhibit strong antimicrobial activity against two main pathogenic bacteria (*P. gingivalis and F. nucleatum*), their clinical translation is constrained by concerns about concentration‐dependent cytotoxicity; notably, cytotoxic effects have been reported at concentrations above 3.7 µg/mL in human fibroblasts [46]. A CS hydrogel co‐delivering antimicrobial peptide‐modified PEG and curcumin‐loaded NPs provided sustained local delivery along with antibacterial, antioxidant, and immunomodulatory effects. In a hypertensive mouse model, the formulation reduced inflammatory cell accumulation and enhanced the antioxidant capacity of macrophages, suggesting its potential for managing periodontal inflammation in the presence of systemic disease [35].

Beyond inorganic NPs, photodynamic therapy (PDT)‐assisted CS nanoplatforms have emerged as promising nonantibiotic approaches. In a recent clinical study, indocyanine green (ICG)‐loaded CS NPs activated using an 810 nm diode laser significantly improved periodontal parameters, including probing pocket depth and clinical attachment level, while reducing *P. gingivalis* and *Aggregatibacter actinomycetemcomitans* when used as an adjunct to scaling and root planing [36].

Despite their strong antibacterial potential, NP‐integrated systems also raise important concerns regarding long‐term cytotoxicity, NP accumulation, biodegradation, and regulatory approval. Their safety and therapeutic performance depend on measurable factors such as NP concentration, particle size, ion‐release rate, duration of tissue exposure, and cumulative local accumulation. However, these parameters are not consistently reported across studies, making meaningful comparisons difficult. Moreover, antibacterial activity in many reported studies has been evaluated against planktonic bacteria rather than mature, matrix‐embedded multispecies biofilms. Therefore, activity against planktonic bacteria may not necessarily translate into effective antibiofilm activity in vivo. Future research should focus on improving the biosafety of these systems while developing multifunctional and responsive platforms capable of simultaneously addressing microbial dysbiosis, inflammation, and periodontal tissue regeneration.

#### 5.1.5 Regenerative and Multifunctional Hydrogel Systems

Regenerative and multifunctional CS‐based hydrogels represent a new class of biomaterials aimed at managing infection, reducing inflammation, angiogenesis, and periodontal tissue regeneration. In contrast to conventional antimicrobial systems, these platforms aim to replicate the dynamic periodontal healing environment by combining bioactive inorganic materials, growth factors, NPs, and controlled‐delivery systems.

Inorganic bioactive materials have been widely incorporated to enhance regenerative performance. Thermosensitive CS hydrogels incorporating β‐tricalcium phosphate exhibit good biocompatibility, structural stability, and cell‐delivery capability, while supporting the viability and osteogenic potential of pre‐osteoblast MC3T3‐E1 cells, indicating their potential for periodontal tissue regeneration [37]. Magnesium oxide‐based CS membranes also exhibit controlled degradation at the end of the 28th day and improved structural stability compared with conventional collagen membranes, indicating their suitability for periodontal bone regeneration applications [17].

Hybrid osteogenic systems that combine hydroxyapatite and bioactive glass can further expand the multifunctional capabilities of CS‐based materials. Amoxicillin‐loaded CS–hydroxyapatite matrices have demonstrated sustained drug release, reduced inflammatory signaling, and improved mechanical integrity, providing both antibacterial and regenerative benefits [22]. Similarly, a multifunctional CS‐poloxamer(407)‐poly(N‐isopropylacrylamide) (PNIPAM) hydrogel incorporating silver NPs and a lipid complex containing quercetin and CAPE showed thermo‐ and pH‐responsive behavior, good injectability, and mechanical stability after injection, while also providing antibacterial activity against *P. gingivalis* [18].

Recent developments have also explored biomimetic multilayer scaffolds that combine structural support with localized therapeutic functions. A dual‐layer CS/alginate/β‐tricalcium phosphate scaffold co‐delivering minocycline and liposome‐encapsulated simvastatin provided sustained antibacterial activity, enhanced osteogenesis, reduced inflammation, and significant alveolar bone regeneration in vivo [38]. Its asymmetric design, consisting of an epithelial barrier layer and a porous osteoconductive layer, was intended to mimic the organization of native periodontal tissues. However, the increased structural complexity of such systems may create challenges related to scalability, reproducibility, and clinical translation. More recently, metal–organic framework (MOF)‐integrated CS hydrogels containing magnesium and gallic acid have demonstrated injectable and self‐healing properties along with dual pH‐ and ROS‐responsive behavior. These systems also showed reduced inflammation, enhanced antibacterial activity, and improved osteogenic potential, highlighting their promise as multifunctional platforms for periodontal tissue repair [47].

These regenerative hydrogels show advancements over conventional localized drug delivery systems, combining antimicrobial, immunomodulatory, angiogenic, and osteogenic functions within a single platform. Yet most of these systems remain in the preclinical stage and often focus on isolated biological mechanisms. Notably, the great majority of the regenerative outcomes reviewed above are alveolar‐bone‐centric; comparatively little attention has been given to coordinated regeneration of the periodontal ligament, cementum, gingival soft tissue, and the periodontal microbiome, even though true periodontal regeneration requires all of these to be restored together, not bone formation alone. This is an important limitation of the current regenerative literature and a priority area for future hydrogel design.

#### 5.1.6 Redox‐Responsive and Smart Adaptive Systems

Stimuli‐responsive CS hydrogels improve localized periodontal therapy by releasing therapeutic agents in response to pathological conditions. Given that periodontitis is characterized by acidic pH, elevated ROS, enzymatic degradation, and persistent inflammation, smart hydrogels engineered to respond to these pathological cues offer substantial therapeutic advantages.

ROS‐responsive systems have received considerable attention due to the central role of oxidative stress in periodontal tissue breakdown. CAPE‐functionalized hydrogels enable ROS‐triggered drug release while suppressing inflammatory cytokine expression by modulating the Nrf2/HO‐1 pathway [14]. Similarly, resveratrol‐derived carbonized polymer dot‐based hydrogels exhibit pH‐responsive degradation, self‐healing, antibacterial activity, and antioxidant effects by regulating the Nrf2/NF‐κB signaling axis [31]. These studies demonstrate increasing interest in microenvironment‐responsive periodontal therapy. In parallel, CS‐coated alginate microspheres enabled sustained metronidazole release while providing ROS scavenging, angiogenic stimulation, and improved wound healing outcomes [26]. Further advancements include dynamically crosslinked, self‐healing hydrogel systems that enhance mechanical adaptability within periodontal defects. Zinc‐ion‐coordinated carboxymethyl CS/oxidized hyaluronic acid hydrogels demonstrated excellent tissue adhesion, fatigue resistance, antimicrobial activity, and osteogenic potential, supporting combined infection control and regenerative functionality [48]. A microenvironment‐responsive CMCS‐oxidized dextran hydrogel delivering embelin exhibited pH‐responsive, self‐healing, and controlled‐release properties, while enhancing antibacterial, immunomodulatory, and osteogenic responses to promote alveolar bone regeneration in experimental periodontitis [49]. More recent work has expanded CS hydrogel therapy toward local immune modulation in metabolically complicated periodontitis. A thermosensitive injectable self‐assembled CS hydrogel co‐loaded with granulocyte‐macrophage colony‐stimulating factor and resveratrol was evaluated as an adjunct to scaling and root planing in a high‐fat diet‐induced rat model of periodontitis. The formulation suppressed inflammatory MAPK/NF‐κB signaling, reduced Th17 Cell infiltration, and increased IL‐10‐associated regulatory immune responses, thereby reducing periodontal inflammation and tissue destruction [50].

Therefore, smart adaptive hydrogels represent a next‐generation approach for periodontal treatment by integrating host‐modulatory and regenerative functions into a responsive platform. However, most of the current systems remain at the proof‐of‐concept stage. Moreover, programmed sequential release systems transitioning from early antimicrobial and antioxidant activity to later‐stage angiogenic and osteogenic stimulation may more effectively replicate the physiological periodontal healing process and improve long‐term regenerative outcomes [27]. A comparative summary of the major therapeutic strategies, their mechanisms, responsiveness, regenerative potential, and limitations is shown in Table 1.

**TABLE 1:** Comparative Analysis of therapeutic strategies in CS‐based systems for periodontal applications.

Class	Representative therapeutic agents	Material type	Evidence level	Major function	Major limitations	Refs.
Antibiotics	Azithromycin	Hydrogel	In vitro	Antibacterial activity and suppression of periodontal pathogens	Antibiotic resistance, burst release, limited long‐term, and clinical validation	[19]
	Doxycycline	Xerogel				[20, 21]
	Amoxicillin	Microparticle				[22]
	Ampicillin	Polyelectrolyte film				[23]
	Minocycline	NP				[24]
	Ciprofloxacin	Nanocomposite hydrogel				[25]
	Metronidazole	Microsphere systems	In vitro/ ex vivo			[15, 26]
	Doxycycline + atorvastatin	Hydrogel	In vitro			[39]
Phytochemicals and natural extracts	Baicalin	Nanohydrogel	In vitro/ in vivo	Antioxidant, anti‐inflammatory , antimicrobial and tissue‐ protective effects	Poor solubility, instability, variable bioactive composition and limited clinical validation.	[28, 29, 41]
	Baicalein	Nanohydrogel				[29]
	Astaxanthin	Hydrogel				[30]
	CAPE	Hydrogel	In vitro			[14]
	Resveratrol	Hydrogel	In vitro/ in vivo			[31]
	*Opuntia ficus‐indica* extract	Hydrogel	In vitro			[42]
	*Salvadora persica* (miswak)	Periodontal chip	Clinical			[43]
	Quercetin + CAPE	Hydrogel	In vitro			[18]
	Curcumin	Hydrogel	In vitro/ in vivo			[35]
Small‐molecule anti‐inflammatory systems	Salicylic acid derivatives	Hydrogel	In vitro/ in vivo	Cytokine suppression and modulation of host inflammatory responses	Limited evidence for sequential or disease‐stage‐specific release; incomplete long‐term validation	[32]
	Acetanilide	Hydrogel	In vitro			[45]
	Acetylsalicylic acid	Hydrogel film				[33]
	Aceclofenac	*in‐situ* gel				[34]
	Atorvastatin	Hydrogel				[39]
NPs and hybrid systems	Nal‐P‐113	Hydrogel	In vitro/ in vivo	Antibacterial activity, ROS modulation, and enhanced regenerative activity	Cytotoxicity, NP accumulation, biodegradation, and regulatory concerns	[27]
	Copper sulfide NPs	Hydrogel				[16]
	Silver NPs	Hydrogel				[18]
	Silver oxide particles	Composite	In vitro			[46]
	Indocyanine green	NP system	Clinical			[36]
	Minocycline + baicalin	Nanohydrogel	In vitro			[28]
Regenerative and multifunctional hydrogel systems	β‐Tricalcium phosphate	Hydrogel	In vitro	Osteogenesis, tissue repair, immunomodulation, and infection control	Structural complexity, scalability, reproducibility, and limited clinical evidence	[37]
	Magnesium oxide	Membrane				[17]
	Hydroxyapatite + amoxicillin	Matrix system				[22]
	Bioactive glass	Hydrogel	In vitro			[45]
	Minocycline + simvastatin	Scaffold	In vitro/ in vivo			[38]
	Magnesium/gallic acid‐modified MOF	Hydrogel	In vitro			[47]
Redox‐responsive and smart adaptive systems	Embelin	Hydrogel	In vitro/ in vivo	Triggered drug release, antioxidant, antimicrobial, and regenerative effects	Single‐trigger design, variable pathological stimuli, and limited long‐term validation	[49]
	Granulocyte‐macrophage colony‐stimulating factor + resveratrol	Hydrogel				[50]
	Zinc ions (Zn^2^^+^)	Hydrogel	In vitro			[48]
	Metronidazole	Microspheres				[26]
	CAPE	Hydrogel				[14]
	Resveratrol‐derived carbonized polymer dots	Hydrogel				[31]
Cell‐derived therapies	Dental pulp stem cell‐derived exosomes	Hydrogel	In vivo	Immunomodulation and periodontal tissue regeneration	Limited number of studies, optimization of loading, stability, and long‐term efficacy	[53]

Abbreviations: CAPE, caffeic acid phenethyl ester; CS, chitosan; ICG, indocyanine green; MOF, metal–organic framework; NP, nanoparticle; ROS, reactive oxygen species; Zn^2^^+^, zinc ion; β‐GP, β‐glycerophosphate; β‐TCP, β‐tricalcium phosphate.

### 5.2 Chitosan Hydrogels as Scaffolds for Cell‐Based and Cell‐Derived Therapies

A distinct and relatively underexplored application of CS hydrogels in periodontal therapy is their use as scaffolds for delivering cell‐derived regenerative components. Their hydrated 3D network can provide a supportive local environment for cell retention, survival, and differentiation. Recent studies in oral regenerative engineering have highlighted the potential of cell‐laden scaffolds, particularly those incorporating periodontal ligament stem cells, for periodontal and maxillofacial tissue reconstruction [51]. Similarly, oral stem cell‐hydrogel systems have been investigated as biomimetic platforms that support cellular localization and provide a 3D environment favorable for cell proliferation, migration, and osteogenic differentiation during periodontal tissue engineering [52].

An important example of cell‐derived therapy involves the incorporation of dental pulp stem cell‐derived exosomes into a CS network. In a mouse model of periodontitis, the exosome‐loaded hydrogel promoted periodontal epithelial and alveolar bone healing while reducing local inflammation. These effects were associated with a shift in macrophage polarization toward an anti‐inflammatory phenotype and were attributed, at least in part, to the delivery of miR‐1246 by the exosomes [53].

Within CS‐based systems, however, the available evidence remains limited. Most reported studies have focused on secretome‐loaded CS hydrogels that deliver stem‐cell‐derived regenerative factors, such as FGF‐2, VEGF, and TGF‐β, in a cell‐free format (Section 5.1.5). In contrast, CS hydrogels incorporating viable cells, cell sheets, or spheroids and specifically validated for periodontal regeneration remain limited. Further research should therefore focus on optimizing CS properties to support cell retention and viability, promote appropriate differentiation, and enable controlled degradation within periodontal defects.

## 6 Drug‐Polymer Chemistry Governing Chitosan Hydrogels

The performance of CS‐based hydrogels is largely influenced by the molecular interactions arising from their reactive amino and hydroxyl groups. These groups participate in ionic interactions, hydrogen bonding, covalent crosslinking, and dynamic supramolecular assembly, which together contribute to the behavior of the hydrogel network [11, 12, 13]. These interactions influence hydrogel stability, swelling, injectability, environmental responsiveness, and drug‐release kinetics within the periodontal microenvironment [13, 14, 15, 22]. In addition to the type of chemical interaction involved, five intrinsic properties of CS are particularly important for hydrogel design.

First, molecular weight affects polymer chain entanglement and solution viscosity. Higher‐molecular‐weight CS generally forms denser and mechanically stronger networks, with slower degradation and more sustained drug release, whereas lower‐molecular‐weight CS tends to produce more permeable and faster‐degrading gels [11, 12, 13]. The degree of deacetylation (DDA) determines the availability of free amino groups for protonation and crosslinking. Therefore, a higher DDA generally increases cationic charge density, mucoadhesive interactions, electrostatic drug complexation, and influence enzymatic degradation [11, 13]. The greater availability of free amino groups may additionally contribute to the antioxidant and anti‐inflammatory properties [12]. The degree of substitution in chemically modified CS derivatives determines the balance between the remaining reactive amino and hydroxyl groups and the newly introduced functional groups, thereby influencing crosslinking capacity and stimuli‐responsive behavior [13, 15, 37]. Polymer concentration affects network density and initial mesh size, with higher concentrations generally producing stiffer gels and slower, more diffusion‐limited drug release, although this may reduce injectability [22, 37]. Finally, crosslinking density, whether achieved through ionic, covalent, or physical interactions, determines the balance between mechanical stability, degradation, and drug‐release rate. Densely crosslinked networks are more resistant to premature erosion and can prolong drug release, but may also restrict swelling and diffusion [22]. Together, these five parameters, rather than any single chemical strategy alone, influence the mucoadhesion, swelling, degradation, drug‐release behavior, and mechanical properties of the resulting hydrogel. Therefore, these characteristics should be clearly reported when comparing different CS‐based hydrogel formulations [11, 12, 13, 22, 37].

Electrostatic interactions are among the most widely used mechanisms in CS‐based systems because the amino groups become protonated when the local pH falls below its pKa (∼6.5). Polyelectrolyte complexes formed between CS and anionic polymers, including hyaluronic acid, alginate, xanthan gum, tragacanth, and carboxymethyl cellulose, can provide tunable swelling, improved mucoadhesion, and sustained drug release [23, 38, 39, 47]. Although increasing the anionic component can generally increase porosity and facilitate diffusion, excessive ionic crosslinking may weaken the hydrogel network and compromise mucoadhesive performance [23, 47].

Dynamic covalent chemistry has further enabled the development of adaptive and stimuli‐responsive hydrogel networks. ROS‐responsive systems containing aryl borate linkages can undergo selective cleavage under oxidative conditions, allowing controlled release of therapeutic agents such as CAPE [14]. Similarly, Schiff base (imine) chemistry between oxidized polysaccharides and amino‐functionalized CS derivatives can produce injectable and self‐healing hydrogels through reversible bond formation. This dynamic behavior allows the material to better adapt to irregular periodontal defects [47]. Non‐covalent interactions, particularly hydrogen bonding and hydrophobic interactions, also contribute to hydrogel network stability and drug encapsulation. Thermoresponsive systems incorporating β‐GP, poly (vinyl alcohol), or glycerol derivatives can undergo sol–gel transitions near physiological temperature through cooperative hydrogen bonding and polymer‐chain rearrangement [15, 45, 47]. These reversible interactions facilitate injection and allow the hydrogel to form in situ following administration.

The incorporation of nanomaterials introduces additional interfacial interactions that can further improve hydrogel functionality. Metal oxide NPs, mesoporous silica, graphene oxide, hydroxyapatite, and bioactive glass can enhance mechanical strength, osteogenic activity, and antibacterial performance through mechanisms involving electrostatic interactions, surface coordination, and ion‐mediated biological signaling [16, 17, 18, 22, 24]. In some systems, these interfacial interactions also contribute to pH‐ or ROS‐responsive drug release, highlighting the importance of nanoscale chemical engineering in the development of multifunctional periodontal hydrogels.

Overall, the therapeutic performance of CS‐based hydrogels is governed by the combined interactions between the drug, CS, and other components of the hydrogel network. Future designs should therefore move toward chemically engineered and responsive networks with improved adaptability to the clinical periodontal environment. Quantitative data on intra‐pocket retention remain limited across the literature. However, where reported, ionically or covalently crosslinked CS systems, such as CS‐alginate and CS‐xanthan hydrogels, generally show longer intra‐pocket retention and slower erosion than simpler thermogelling systems [38]. In contrast, thermosensitive β‐GP‐based gels typically achieve gelation within seconds to a few minutes at body temperature but may show comparatively shorter structural persistence [15]. More systematic quantitative comparisons of retention time, erosion rate, gelation time, and overall drug‐release duration across different formulation types are therefore needed. Such comparisons would provide a stronger basis for determining whether experimental CS hydrogels offer meaningful improvements over existing intra‐pocket drug‐delivery systems. The major chemical interactions governing CS hydrogel performance and their associated functional outcomes are summarized in Table 2.

**TABLE 2:** Drug‐polymer chemistry and functional interactions in CS‐based hydrogels.

Chemical strategy	Interaction type	Functional outcome	Therapeutic systems	Major advantage	Refs.
Polyelectrolyte complexation	Electrostatic interactions between protonated CS and anionic polymers	Controlled swelling and drug diffusion	CS‐hyaluronic acid‐based delivery systems	Tunable drug diffusion and mucoadhesion	[23]
Ionic crosslinking	Ionic interactions between CS and multivalent/anionic components	Network stabilization and controlled degradation	CS‐containing ionic hydrogel systems	Improved structural stability and retention	[39, 42]
Dynamic covalent crosslinking	Reversible covalent bonds, including Schiff‐base chemistry	Self‐healing and injectability	Dynamically crosslinked CS‐based hydrogels	Adaptability to irregular defects	[16, 47, 48, 49]
Boronate/diselenide redox chemistry	ROS‐sensitive covalent or dynamic linkages	ROS‐triggered release and antioxidant activity	CAPE‐ and diselenide‐crosslinked CS hydrogels	Responsiveness to oxidative stress	[14, 30]
β‐GP thermogelation	Hydrogen bonding and temperature‐dependent polymer interactions	Sol‐gel transition near physiological temperature	Thermosensitive CS/β‐GP hydrogels	Injectable in situ gel formation	[15, 27, 34]
NP‐polymer interactions	Electrostatic, coordination, and interfacial interactions	Enhanced antibacterial, mechanical, and regenerative properties	CuS‐, Ag‐, silica‐, and HNT‐containing CS hydrogels	Multifunctional performance	[16, 18, 25, 27, 28, 34]
Hydrogen‐bonding/supramolecular interactions	Reversible non‐covalent interactions	Mechanical reinforcement and network stability	Composite CS hydrogel systems	Improved mechanical adaptability	[18, 45]
Polymer‐mineral interactions	Interfacial interactions between CS and calcium‐phosphate/mineral phases	Osteogenic support and structural reinforcement	CS‐hydroxyapatite/β‐TCP systems	Improved regenerative potential	[22, 37]

Abbreviations: CAPE, caffeic acid phenethyl ester; CS, chitosan; HNT, halloysite nanotube; NP, nanoparticle; ROS, reactive oxygen species; β‐GP, β‐glycerophosphate; β‐TCP, β‐tricalcium phosphate.

## 7 Summary and Perspectives

CS‐based hydrogels have emerged as promising multifunctional platforms for periodontal therapy because of their intrinsic mucoadhesiveness, biocompatibility, injectability, and ability to provide sustained local drug delivery [7, 12]. The literature reviewed in this work demonstrates growing interest in hydrogel systems designed to address several aspects of periodontitis simultaneously, including bacterial infection, inflammation, oxidative stress, tissue destruction, and regeneration.

Compared with non‐hydrogel CS systems such as NPs, microparticles, films, xerogels, membranes, and chips [19, 20, 21, 22, 23, 24, 33, 36, 41, 43, 46] discussed in this review, hydrogels offer a distinct advantage with respect to drug delivery by combining a hydrated 3D network with injectability, mucoadhesion, and conformal filling of irregular periodontal pockets. This also provides a versatile platform for incorporating multiple therapeutic components, integrating antimicrobial, anti‐inflammatory, antioxidant, and regenerative functions within the same formulation [14, 15, 16, 17, 18, 25, 27, 30, 31, 37, 38, 42]. However, non‐hydrogel systems may provide greater structural and mechanical stability, easier handling, and more established clinical applications [23, 33, 36, 43]. Hydrogels, in contrast, remain limited by relatively low mechanical strength, formulation‐dependent gelation and degradation, possible premature drug release, challenges in sterilization, storage stability, and reproducibility [7, 12]. Thus, the main advantage of CS hydrogels lies not in replacing all non‐hydrogel systems, but in their ability to provide an adaptable local microenvironment that integrates drug delivery with multifunctional and potentially regenerative effects.

A major trend is the increasing diversity of therapeutic agents incorporated into CS‐based systems, including antibiotics, phytochemicals, NPs, growth factors, and redox‐active molecules [5, 8, 9, 10, 11, 27, 39]. This shift reflects a broader understanding of periodontitis as a multifactorial disease involving microbial dysbiosis, oxidative stress, immune dysregulation, and progressive destruction of the periodontal supporting tissues [1, 2, 3, 4]. As a result, therapeutic approaches focused solely on bacterial elimination may not be sufficient to achieve sustained clinical improvement. In this context, NP‐assisted and PDT‐based approaches represent a growing interest in nonantibiotic and locally activated antimicrobial strategies. These systems offer the possibility of generating ROS‐mediated bacterial killing without relying exclusively on conventional antibiotics. However, factors such as treatment conditions, light penetration and delivery, and the complex nature of periodontal biofilms remain important challenges for the clinical application of PDT‐based approaches [36].

Another important challenge is the limited ability of many current systems to penetrate and disrupt mature multispecies biofilms. Although numerous studies report strong antimicrobial activity against planktonic bacteria, fewer have evaluated these systems against mature biofilms containing dense extracellular polymeric substances, which more closely resemble the clinical periodontal environment [27, 28]. This gap highlights the need for next‐generation hydrogel systems that incorporate biofilm‐disrupting enzymes, charge‐switching properties, or matrix‐targeting mechanisms to improve penetration and therapeutic activity under physiological conditions.

Stimuli‐responsive hydrogel systems enable localized and controlled therapeutic release, demonstrating responsiveness to pH, ROS, temperature, and inflammatory signals [14, 15, 30, 31, 32, 38]. In particular, ROS‐responsive and self‐healing hydrogels are relevant given the significant role of oxidative stress in periodontal tissue destruction. A comparative summary of stimuli‐responsive CS hydrogel systems and their therapeutic advantages is listed in Table 3, which classifies each system by its primary trigger mechanism as reported by the original authors; systems that are explicitly designed and validated for more than one simultaneous stimulus are listed under “Multi‐stimuli responsive”. However, most existing systems rely on single‐stimulus responsiveness, despite the dynamic nature of periodontal microenvironments. Because periodontitis involves interacting microbial, inflammatory, enzymatic, and oxidative changes, future designs should aim to achieve appropriate multifunctionality without introducing unnecessary structural complexity.

**TABLE 3:** Comparative analysis of stimuli‐responsive CS‐based systems.

Stimulus type	Trigger mechanism	Therapeutic advantage	Therapeutic systems	Limitation	Refs.
pH‐	Acidic periodontal microenvironment alters CS protonation, swelling, or responsive linkages	Site‐specific and accelerated therapeutic release	pH‐responsive CS‐containing drug‐delivery systems	Periodontal pH varies spatially and temporally; a single‐trigger response may be insufficient	[15, 25, 27, 31]
ROS‐	Elevated oxidative stress cleaves or alters ROS‐sensitive linkages	Targeted antioxidant, anti‐inflammatory, and controlled‐release activity	CAPE‐conjugated and diselenide‐crosslinked CS hydrogels	Variable ROS concentrations in vivo	[14, 30, 31]
Temperature‐	Temperature‐dependent sol‐gel transition	Injectable in situ gel formation and improved local retention	β‐GP‐containing CS systems; thermosensitive CS nanocomposite hydrogels	Potential mechanical/structural instability after gelation	[15, 18, 27, 34, 42]
Light‐	External light activates photosensitizer‐mediated ROS generation	Localized photodynamic antibacterial activity	ICG‐loaded CS NP system	Requires an external light source and adequate light penetration	[36]
Multi‐stimuli	A combination of two or more pathological/environmental signals	More adaptive therapeutic release	pH/ROS‐responsive and multifunctional CS‐based systems	Increased formulation complexity and manufacturing challenges	[31, 47, 48, 49, 50]
Self‐healing/dynamic systems	Reversible dynamic bonds reform after mechanical disruption	Structural recovery and adaptability to periodontal defects	Dynamically crosslinked and ion‐coordinated CS‐containing hydrogels	Limited long‐term mechanical and biological validation	[16, 30, 48, 49, 50]

Abbreviations: CAPE, caffeic acid phenethyl ester; CS, chitosan; ICG, indocyanine green; Multi‐stimuli systems respond to ≥2 stimuli; self‐healing/dynamic systems exhibit reversible network reformation; NP, nanoparticle; ROS, reactive oxygen species; β‐GP, β‐glycerophosphate

Few studies have directly compared CS‐based hydrogels with clinically established periodontal delivery systems such as Atridox (doxycycline gel), PerioChip (chlorhexidine chip), and Arestin (minocycline microspheres) [6]. These products provide useful clinical benchmarks, with reported local drug‐release/retention periods of approximately 7–14 days for Atridox, 7–10 days for PerioChip, and 14–21 days for Arestin, together with demonstrated periodontal clinical outcomes [6]. In comparison, several CS‐based systems have shown prolonged local drug release and multifunctional activity. Notably, the thermosensitive CS/polydopamine‐Nal‐P‐113 hydrogel provided approximately 13 days of release and produced about 99% bacterial reduction, approaching the release duration reported for established products; however, its efficacy has been demonstrated only in vitro and in a rodent model [27]. The Salvadora persica CS chip is currently one of the few systems with human clinical evidence showing improvements in probing pocket depth, bleeding on probing, and clinical attachment level; however, its retention time and release duration were not quantitatively established [43]. CS hydrogels may also offer practical advantages through injectable administration, sustained local delivery, and incorporation of multiple therapeutic or regenerative functions [7, 16, 27]. Nevertheless, evidence of superior handling, retention, safety, or clinical efficacy relative to approved products remains insufficient, as direct head‐to‐head clinical comparisons are scarce. Future studies should therefore evaluate CS systems using standardized clinical endpoints and directly compare retention, release kinetics, handling, safety, and therapeutic efficacy with existing local‐delivery products.

Beyond their therapeutic potential, the safety and biological consequences of complex CS‐based systems require careful consideration. NP‐ and inorganic‐component‐containing systems may offer enhanced antibacterial and regenerative performance, but their long‐term safety, degradation behavior, potential accumulation, and regulatory implications require careful evaluation [17, 25, 28, 37]. The preservation of beneficial oral microbiota should also be considered when developing antimicrobial systems. Furthermore, regenerative strategies have predominantly focused on alveolar bone repair, with comparatively limited attention given to gingival tissue regeneration. Addressing these aspects will be important for developing multifunctional systems that provide therapeutic benefits without compromising long‐term tissue and microbiome homeostasis.

Despite rapid progress toward multifunctional and stimuli‐responsive systems, several barriers continue to limit clinical translation. Considerable variation in CS molecular weight, degree of deacetylation, crosslinking chemistry, drug loading, and experimental protocols makes results difficult to reproduce and compare across studies [7, 27, 45]. Moreover, many investigations rely on simplified in vitro models or limited animal models that do not fully reproduce salivary flow, masticatory forces, multispecies biofilms, immune responses, and enzymatic degradation within the periodontal pocket [5, 27, 39]. Greater use of clinically relevant multispecies biofilm models and standardized preclinical outcome measures would improve the translational value of these studies.

From a translational perspective, factors such as sterilization, storage stability, batch‐to‐batch consistency, scalability, and regulatory classification should be addressed early during formulation development rather than only after proof‐of‐concept studies. Injectable systems containing peptides, natural products, NPs, or dynamic crosslinks may be particularly sensitive to sterilization procedures and prolonged storage. Future studies should therefore place greater emphasis on standardized multispecies biofilm models, long‐term safety evaluation, stability and sterilization testing, assessment under clinically relevant gingival crevicular fluid and mechanical conditions, and direct comparison with established periodontal therapies [7, 27, 45]. More advanced approaches, including personalized and theranostic hydrogels, offer promising long‐term possibilities, but their clinical realization will require substantial advances in manufacturing, regulatory approval, and clinical validation.

Overall, the next phase of CS hydrogel development should not focus simply on increasing formulation complexity. Instead, the goal should be to achieve a practical balance between multifunctionality, safety, reproducibility, manufacturability, and therapeutic performance. Moving beyond laboratory proof‐of‐concept studies toward standardized preclinical evaluation and well‐designed human clinical trials will be essential to determine whether CS‐based hydrogels can provide meaningful clinical advantages over currently available local periodontal drug‐delivery systems.

## 8 Conclusions

CS‐based hydrogels are promising materials for localized periodontal treatment owing to their biocompatibility, adhesiveness, and controlled drug‐release behavior. Recent research has focused on multifunctional and responsive hydrogels that help reduce infection, inflammation, and oxidative stress while supporting tissue regeneration. The addition of NPs, phytochemicals, and other bioactive agents has further improved their therapeutic potential. However, clinical use remains limited due to challenges such as a lack of standardization, limited long‐term safety data, and insufficient preclinical models that mimic the oral environment. Future research should focus on developing safer, more effective, and clinically applicable CS‐based hydrogels for improved periodontal therapy.

## Author Contributions

**Vikesh Kumar** and **B.T. Nandish**: conceptualization. **Vikesh Kumar**: literature search and study selection. **Vikesh Kumar** and **B.T. Nandish**: screening of records. **Vikesh Kumar** and **B.T. Nandish**: resolution of discrepancies. **Sowmya Rao** and **Manojna K.M**.: critical appraisal and synthesis of the literature. **Vikesh Kumar**: writing – original draft preparation. **Vikesh Kumar, B.T. Nandish, Sowmya Rao**, and **Manojna K.M**.: writing – review and editing. **B.T. Nandish**: supervision. All authors have read and agreed to the published version of the manuscript.

## Conflicts of Interest

The authors declare no conflicts of interest.

## Use of Generative AI and AI‐Assisted Technologies in the Writing Process

During the preparation of this manuscript, the authors used ChatGPT (accessed in September 2026) for language refinement and grammar correction. After its use, the authors thoroughly reviewed, verified, and revised all AI‐assisted content to ensure accuracy and originality. The authors take full responsibility for the integrity and final content of the published article.

## Data Availability

The data that support the findings of this study are available from the corresponding author upon reasonable request.

## References

[1] Y. Deng , J. Xiao , L. Ma , et al., “Mitochondrial Dysfunction in Periodontitis and Associated Systemic Diseases: Implications for Pathomechanisms and Therapeutic Strategies,” International Journal of Molecular Sciences 25, no. 2 (2024): 1024, 10.3390/ijms25021024. PMC10816612 38256098

[2] S. S. Socransky and A. D. Haffajee , “Microbial Mechanisms in the Pathogenesis of Destructive Periodontal Diseases: A Critical Assessment,” Journal of Periodontal Research 26, no. 3 (1991): 195–212, 10.1111/j.1600-0765.1991.tb01646.x. 1831843

[3] A. Basic and G. Dahlén , “Microbial Metabolites in the Pathogenesis of Periodontal Diseases: A Narrative Review,” Frontiers in Oral Health 4 (2023): 1210200, 10.3389/froh.2023.1210200. PMC10300593 37388417

[4] P. Dahiya , R. Kamal , R. Gupta , R. Bhardwaj , K. Chaudhary , and S. Kaur , “Reactive Oxygen Species in Periodontitis,” Journal of Indian Society of Periodontology 17, no. 4 (2013): 411, 10.4103/0972-124X.118306. PMC3800399 24174716

[5] C.‐M. Radu , C. Radu , E.‐M. Arbănaşi , et al., “Exploring the Efficacy of Novel Therapeutic Strategies for Periodontitis: A Literature Review,” Life 14, no. 4 (2024): 468, 10.3390/life14040468. PMC11050937 38672739

[6] M. Fayazi , M. Rostami , M. Amiri Moghaddam , et al., “A state‐of‐the‐art Review of the Recent Advances in Drug Delivery Systems for Different Therapeutic Agents in Periodontitis,” Journal of Drug Targeting 33, no. 5 (2025): 612–647, 10.1080/1061186X.2024.2445051. 39698877

[7] M. Vigata , C. Meinert , D. W. Hutmacher , and N. Bock , “Hydrogels as Drug Delivery Systems: A Review of Current Characterization and Evaluation Techniques,” Pharmaceutics 12, no. 12 (2020): 1188, 10.3390/pharmaceutics12121188. PMC7762425 33297493

[8] H. Sun , J. Luan , and S. Dong , “Hydrogels Promote Periodontal Regeneration,” Frontiers in Bioengineering and Biotechnology 12 (2024): 1411494, 10.3389/fbioe.2024.1411494. PMC11140061 38827033

[9] M. El‐Nablaway , F. Rashed , E. S. Taher , et al., “Bioactive Injectable Mucoadhesive Thermosensitive Natural Polymeric Hydrogels for Oral Bone and Periodontal Regeneration,” Frontiers in Bioengineering and Biotechnology 12 (2024): 1384326, 10.3389/fbioe.2024.1384326. PMC11166210 38863491

[10] S. Ran , L. Xue , X. Wei , et al., “Recent Advances in Injectable Hydrogel Therapies for Periodontitis,” Journal of Materials Chemistry B 12, no. 25 (2024): 6005–6032, 10.1039/d3tb03070a. 38869470

[11] L. Qiqi , D. Wang , C. Xiao , H. Wang , and S. Dong , “Advances in Hydrogels for Periodontitis Treatment,” ACS Biomaterials Science & Engineering 10 (2024): 2742–2761, 10.1021/acsbiomaterials.4c00220. 38639082

[12] R. Biswas , S. Mondal , M. A. Ansari , et al., “Chitosan and Its Derivatives as Nanocarriers for Drug Delivery,” Molecules 30, no. 6 (2025): 1297, 10.3390/molecules30061297. PMC11946192 40142072

[13] I. S. Bayer , “Recent Advances in Mucoadhesive Interface Materials, Mucoadhesion Characterization, and Technologies,” Advanced Materials Interfaces 9 (2022): 2200211, 10.1002/admi.202200211.

[14] G. Wang , Y. Wang , S. Li , et al., “ROS‐responsive Caffeic Acid Phenethyl Ester‐conjugated Carboxymethyl Chitosan Hydrogel for Treatment of Periodontitis,” Journal of Drug Delivery Science and Technology 101 (2024): 106255, 10.1016/j.jddst.2024.106255.

[15] N. M. Maddeppungeng , N. A. Syahirah , N. Hidayati , et al., “Specific Delivery of Metronidazole Using Microparticles and Thermosensitive In Situ Hydrogel for Intrapocket Administration as an Alternative in Periodontitis Treatment,” Journal of Biomaterials Science, Polymer Edition 35, no. 11 (2024): 1726–1749, 10.1080/09205063.2024.2349414, Polymer edition. 38769614

[16] Y. Yang , C. Xu , S. Xu , et al., “Injectable Hydrogels Activated With Copper Sulfide Nanoparticles for Enhancing Spatiotemporal Sterilization and Osteogenesis in Periodontal Therapy,” Biomaterials Science 13, no. 6 (2025): 1434–1448, 10.1039/d3bm02134c. 38711336

[17] J. Harris and K. Gurumoorthy , “Development and Characterization of Novel Magnesium Oxide Nanoparticle‐impregnated Chitosan‐based Guided Tissue Regeneration Membrane—An In Vitro Study,” Journal of Indian Society of Periodontology 28, no. 5 (2025): 522–528, 10.4103/jisp.jisp_554_23. PMC11932556 40134407

[18] C. Zhang , Y. Fei , M. Li , et al., “Chitosan‐P407‐PNIPAM Hydrogel Loaded With AgNPs/Lipid Complex for Antibacterial, Inflammation Regulation and Alveolar Bone Regeneration in Periodontitis Treatment,” International journal of biological macromolecules 307 (2025): 142080, 10.1016/j.ijbiomac.2025.142080. 40107529

[19] J. Kwiatek , M. Paczkowska‐Walendowska , A. Rył , et al., “Azithromycin‐loaded Nanoparticles Incorporated in Chitosan‐based Soft Hydrogels: A Novel Approach for Dental Drug Delivery,” Pharmaceutics 17, no. 3 (2025): 304, 10.3390/pharmaceutics17030304. PMC11945840 40142968

[20] S. Longre , D. Rana , S. Rangra , et al., “Quality‐by‐Design Based Development of Doxycycline Hyclate–Loaded Polymeric Microspheres for Prolonged Drug Release,” Aaps Pharmscitech [Electronic Resource] 25, no. 3 (2024): 49, 10.1208/s12249-024-02760-7. 38424393

[21] F. Zafar , M. A. Sheraz , S. A. Ali , M. Riaz , S. Ahmed , and Z. Anwar , “Novel Antibiotic‐loaded Pegylated Xerogels of Acidified Chitosan for Periodontal Diseases,” Current Drug Delivery 23, no. 8 (2026): 945–965, 10.2174/0115672018377472250326061736. 40148301

[22] B. Zhang , S. Yan , Y. Zhang , and Y. Wu , “Optimizing Locally Delivered Periodontitis Therapy: Development of Chitosan‐Hydroxyapatite‐Encapsulated Drug via Electrospraying,” Journal of Applied Polymer Science 142, no. 1 (2025): e56309, 10.1002/app.56309.

[23] S. Arısoy , K. Bux , R. Herwig , and E. Şalva , “Development, Evaluation, and Molecular Dynamics Study of Ampicillin‐Loaded Chitosan–Hyaluronic Acid Films as a Drug Delivery System,” ACS omega 9, no. 18 (2024): 19805–19815, 10.1021/acsomega.3c08076. PMC11079874 38737032

[24] J. Yang , J. Mou , K. Ding , et al., “A Biodegradable Nano‐drug Delivery Platform for Co‐delivery of Minocycline and Chitosan to Achieve Efficient and Safe Non‐surgical Periodontitis Therapy,” Current Drug Delivery 22, no. 8 (2025): 1143–1156, 10.2174/0115672018305286240502060504. 38706352

[25] S. Zare , M. Eskandani , S. Vandghanooni , H. Hossainpour , and M. Jaymand , “Ciprofloxacin‐loaded Chitosan‐based Nanocomposite Hydrogel Containing Silica Nanoparticles as a Scaffold for Bone Tissue Engineering Application,” Carbohydrate Polymer Technologies and Applications 7 (2024): 100493, 10.1016/j.carpta.2024.100493.

[26] N. A. Mohd Zayi , M. L. Mohamed Halim , A. F. Harun Ismail , and M. Y. Mohamad , “ *In Vitro* Analysis of Collagen‐chitosan Scaffold Loaded With Metronidazole Nanoparticle for Periodontal Disease Treatment,” Sustainable & Green Materials 1, no. 1 (2025): 17–27, 10.1080/29965292.2024.2425443.

[27] S. Ma , X. Lu , X. Yu , et al., “An Injectable Multifunctional Thermo‐sensitive Chitosan‐based Hydrogel for Periodontitis Therapy,” Biomaterials Advances 142 (2022): 213158, 10.1016/j.bioadv.2022.213158. 36288629

[28] J. Haining , “Antibacterial and Immunomodulatory Chitosan Nanohydrogels for Periodontitis Therapy,” International Dental Journal 75 (2025): 105144, 10.1016/j.identj.2025.105144.

[29] L. Guo , M. Han , H. Zhang , and Y. Han , “Effect of Chitosan/Dioleyl Phosphatidyl Ethanolamine‐Baicalein Nanohydrogel in the Treatment of Rat With Periodontitis,” Heliyon 10, no. 3 (2024): e25209, 10.1016/j.heliyon.2024.e25209. PMC10864910 38356511

[30] Y. Wu , C. Chen , S. Lu , et al., “Tailored Modulation of Reactive Oxygen Species and Anti‐inflammatory Responses in Periodontitis Using In Situ Diselenium‐crosslinked Chitosan Hydrogel,” Nano Today 61 (2025): 102588, 10.1016/j.nantod.2024.102588.

[31] N. Li , X. Yan , M. Cheng , et al., “Construction of a Microenvironment‐Responsive Antibacterial Hydrogel Packed with Immunomodulatory Carbonized Polymer Dots for Periodontitis Treatment from Multiple Perspectives,” Advanced Functional Materials 35, no. 22 (2025): 2418407, 10.1002/adfm.202418407.

[32] J. Zhang , L. Meng , Y. Jia , J. Li , X. Xu , and X. Xu , “Development of an Injectable Salicylic Acid–choline Eutectic Hydrogel for Enhanced Treatment of Periodontitis,” Materials Horizons 12, no. 11 (2025): 3788–3802, 10.1039/d4mh01563k. 40052257

[33] J. Zhang , L. Wang , Y. Mi , F. Dong , and Z. Guo , “Preparation and Properties of Crosslinked Quaternized Chitosan‐based Hydrogel Films Ionically Bonded With Acetylsalicylic Acid for Biomedical Materials,” Marine Drugs 22, no. 10 (2024): 450, 10.3390/md22100450. PMC11509478 39452858

[34] S. Singha , M. S. Chellathurai , S. Mahmood , et al., “Thermosensitive in‐situ Gel Based on Halloysite Nanotube/Chitosan for Site‐specific Delivery of Aceclofenac,” Journal of Pharmaceutical Sciences 115 (2026): 104228, 10.1016/j.xphs.2026.104228. 41771486

[35] S. Xu , B. Hu , T. Dong , et al., “Alleviate Periodontitis and Its Comorbidity Hypertension Using a Nanoparticle‐Embedded Functional Hydrogel System,” Advanced Healthcare Materials 12, no. 20 (2023): 2203337, 10.1002/adhm.202203337. 36972711

[36] P. Shashikumar , R. L. R , R. S , S. K. Gujjari , and S. Nisha , “Evaluation of Photodynamic Therapy Using Indocyanine Green Loaded Chitosan Nanoparticles, as an Adjunct to Non‐Surgical Periodontal Therapy in Patients With Stage I and II Periodontitis″. A Randomized Controlled Trial,” Photodiagnosis and Photodynamic Therapy 58 (2026): 105409, 10.1016/j.pdpdt.2026.105409. 41720202

[37] N. Tan , M. Sabalic‐Schoener , L. Nguyen , and F. D'Aiuto , “β‐Tricalcium Phosphate‐loaded Chitosan‐based Thermosensitive Hydrogel for Periodontal Regeneration,” Polymers 15, no. 20 (2023): 4146, 10.3390/polym15204146. PMC10611029 37896389

[38] M. Wei , D. Wang , D. Li , et al., “Bioengineered Bilayer Scaffold With Dual‐Modal Delivery for Alveolar Bone Regeneration in Periodontitis Therapy,” Glycoscience & Therapy 2 (2026): 100044, 10.1016/j.glycos.2026.100044.

[39] B. Yılmaz , Y. Yıldırım , N. Yakar , G. Özdemir , A. Kantarci , and G. Emingil , “Dual‐drug Carboxymethyl Chitosan Hydrogel: Development, Characterization, and In Vitro Evaluation for Periodontal Therapy,” Carbohydrate polymers 363 (2025): 123726, 10.1016/j.carbpol.2025.123726. 40441835

[40] W. Li , M. Li , T. Cai , Y. Ding , W. Tian , and S. Guo , “Intelligent Biomaterials for Periodontitis and Peri‐implantitis Therapies: Stimuli‐responsive Strategies Targeting Inflammation and Regeneration,” Journal of Materials Chemistry B 13, no. 47 (2025): 15167–15189, 10.1039/d5tb01852h. 41190472

[41] M. Paczkowska‐Walendowska , I. Koumentakou , M. Lazaridou , et al., “3D‐printed Chitosan‐based Scaffolds With *Scutellariae baicalensis* Extract for Dental Applications,” Pharmaceutics 16, no. 3 (2024): 359, 10.3390/pharmaceutics16030359. PMC10974774 38543253

[42] R. Conte , A. Valentino , I. De Luca , et al., “Thermo‐responsive Hydrogel Containing Microfluidic Chitosan Nanoparticles Loaded With *Opuntia Ficus‐indica* Extract for Periodontitis Treatment,” International Journal of Molecular Sciences 25, no. 17 (2024): 9374, 10.3390/ijms25179374. PMC11395269 39273327

[43] F. H. Al‐Bayaty , A. A. Kamaruddin , M. A. Ismail , and M. M. J. Al‐Obaidi , “Evaluation of Clinical Efficacy of Biodegradable Chip Containing *Salvadora persica* Extract in Chitosan Base as an Adjunct to Scaling and Root Planning in the Management of Periodontitis,” Sultan Qaboos University Medical Journal 24, no. 3 (2024): 360, 10.18295/squmj.6.2024.028. PMC11370949 39234331

[44] X. Chen , N. Huang , D. Wang , et al., “Sulfated Chitosan‐modified CuS Nanocluster: A Versatile Nanoformulation for Simultaneous Antibacterial and Bone Regenerative Therapy in Periodontitis,” ACS nano 18, no. 22 (2024): 14312–14326, 10.1021/acsnano.4c00137. 38767151

[45] S. Sajjad , O. Görke , F. Z. Kocak , et al., “Acetanilide‐Loaded Injectable Hydrogels With Enhanced Bioactivity and Biocompatibility for Potential Treatment of Periodontitis,” Journal of Applied Polymer Science 141, no. 14 (2024): e55200, 10.1002/app.55200.

[46] N. Nasrabadi , N. Ramezanian , P. Ghorbanian , A. Forouzanfar , and H. S. Mohammadipour , “Evaluation of Cytotoxicity and Antimicrobial Activity of Experimental Composites Containing Chitosan‐silver Oxide Particles Against Two Main Pathogenic Bacteria in Periodontal Disease,” Protein & Peptide Letters 31, no. 2 (2024): 97–106, 10.2174/0109298665240242231016103321. 37921156

[47] R. Sacourbaravi , Z. Ansari‐Asl , E. Hoveizi , and E. Darabpour , “Poly(vinyl alcohol)/Chitosan Hydrogel Containing Gallic Acid‐Modified Fe, Cu, and Zn Metal–Organic Frameworks (MOFs): Preparation, Characterization, and Biological Applications,” ACS Applied Materials & Interfaces 16, no. 45 (2024): 61609–61620, 10.1021/acsami.4c11053. 39476423

[48] M. Yang , D. Du , Y. Hao , Z. Meng , H. Zhang , and Y. Liu , “Preparation of an Injectable Zinc‐containing Hydrogel With Double Dynamic Bond and Its Potential Application in the Treatment of Periodontitis,” RSC advances 14, no. 27 (2024): 19312–19321, 10.1039/d4ra00546e. PMC11181151 38887645

[49] G. Cai , L. Ren , J. Yu , et al., “A Microenvironment‐Responsive, Controlled Release Hydrogel Delivering Embelin to Promote Bone Repair of Periodontitis via Anti‐Infection and Osteo‐Immune Modulation,” Advanced science 11, no. 34 (2024): 2403786, 10.1002/advs.202403786. PMC11425865 38978324

[50] Y. Zhu , A. Ali , G. Mulinari dos Santos , et al., “A Chitosan‐based Hydrogel to Modulate Immune Cells and Promote Periodontitis Healing in the High‐fat Diet‐induced Periodontitis Rat Model,” Acta Biomaterialia 200 (2025): 452–463, 10.1016/j.actbio.2025.05.034. 40379118

[51] A. Abdeen , E. M. Elmorsy , H. F. Hetta , et al., “Evolution of Periodontal Ligament Stem Cells and Their Laden Scaffolds as Game Changers in Oral and Maxillofacial Reconstruction,” Tissue and Cell 200 (2026): 103895, 10.1016/j.tice.2026.103895. 42679616

[52] N. Taymour , M. Alkandari , M. Alkandari , et al., “Oral Stem Cells in Combination With Hydrogels as Biomimetic Bioactive Platforms for Periodontal Tissue Engineering,” Frontiers in Oral Health 6 (2025): 1740392, 10.3389/froh.2025.1740392. PMC12920563 41727277

[53] Z. Shen , S. Kuang , Y. Zhang , et al., “Chitosan Hydrogel Incorporated With Dental Pulp Stem Cell‐derived Exosomes Alleviates Periodontitis in Mice via a Macrophage‐dependent Mechanism,” Bioactive Materials 5, no. 4 (2020): 1113–1126, 10.1016/j.bioactmat.2020.07.002. PMC7371600 32743122

